# Effects of the MR-DTI Characteristics of the Trigeminal Ganglion Target on Radiofrequency Treatment in Patients with Trigeminal Neuralgia: A Retrospective Observational Clinical Study

**DOI:** 10.1155/2023/1988926

**Published:** 2023-01-17

**Authors:** Xu Su, Zhengming Wang, Min Cheng, Yu Tian, Chao Du

**Affiliations:** ^1^Departments of Neurosurgery, China-Japan Union Hospital of Jilin University, Changchun, Jilin 130033, China; ^2^Departments of Radiation, China-Japan Union Hospital of Jilin University, Changchun, Jilin 130033, China

## Abstract

**Background:**

In the percutaneous treatment of trigeminal neuralgia (TN), the difficulty in accessing the foramen ovale (FO) has been widely recognized. However, the most efficient percutaneous treatment target is the so-called trigeminal ganglion target (TGT). We propose that the TGT in a puncture can be identified by magnetic resonance diffusion tensor imaging (MR-DTI).

**Objectives:**

To observe the effect of the characteristics of the TGT as detected by MR-DTI on percutaneous stereotactic radiofrequency rhizotomy (PSR) in TN patients.

**Methods:**

In our observational study, we preoperatively performed MR-DTI and/or 3D-CT for 48 TN patients, analyzed the characteristics of the TGT and/or FO, and designed appropriate surgical schemes for producing an accurate PSR trajectory according to these characteristics. The position and size of the TGT aided in adjusting the puncture angle and guiding the approach. Then, we successfully performed a customized PSR guided by the characteristics of the FO or TGT. During the postoperative and follow-up periods, we assessed the effect of treatment with pain scores and MR-DTI findings.

**Results:**

The characteristics of the TGT vary from patient to patient. We performed PSR with a single puncture guided by MR-DTI and 3D-CT in 16 patients, and only one patient required three punctures. All three of these punctures reached the FO target, as confirmed by intraoperative C-arm X-ray. We finally reached the TGT successfully after 2 additional attempts, confirming that the probe reached the TGT that accurately covered the pain territory with an electrophysiology test. The characteristics of the TGT were negatively correlated with the number of PSR punctures. Fewer complications occurred for PSRs guided by the TGT than for PSRs guided by the FO.

**Conclusions:**

The characteristics of the TGT are correlated with the number of punctures in the PSR. The application of MR-DTI for detecting the size of the TGT is an important step in predicting the difficulty of puncture. The PSR approach can be guided by the TGT and FO for TN patients who present with multiple adverse factors and thus may be beneficial in reducing the number of complications.

## 1. Introduction

Trigeminal neuralgia (TN) is a neurologic disorder of the trigeminal nerve (cranial nerve V, CNV) affecting 3– 27 per 100,000 people [1, 2]. Percutaneous stereotactic radiofrequency rhizotomy (PSR) is an important therapy that involves puncturing the trigeminal ganglion (TG) through the foramen ovale (FO) [1, 3–6].

The single-puncture rate in radiofrequency treatment has attracted great attention. The PSR procedure has two key steps, including puncture of the FO and TG [7]. In certain circumstances, the FO is difficult to access in certain circumstances, such as when the trajectory is obstructed by the maxilla or mandible, the distance between the mandible and tooth ridge is too narrow, the FO is covered by the peripheral bones, or the FO is small; in these circumstances, the first puncture attempt is often unsuccessful, and multiple attempts are needed [8, 9], potentially causing unnecessary damage and complications. However, inaccurate localization is the primary reason for puncture failure and is considered a significant cause of pain recurrence and complications. Accordingly, accurate puncture is of the utmost importance, as it may decrease the incidence of complications and improve the effects of treatment. The difficulty in accessing the FO can be analyzed and lessened by methods such as three-dimensional computed tomography (3D-CT) imaging.

The efficient therapeutic target of PSR is a part of the TG, known as the trigeminal ganglion target (TGT). Conventional imaging is unable to identify and locate this part of the TG, which controls the affected CNV branches [4, 10]. It has been reported that magnetic resonance diffusion tensor imaging (MR-DTI) can detect CNV lesions in TN patients [11–15]. The success of treatment with radiofrequency is directly related to the area affected by the lesion [6]. Confirmation of probe insertion into the TGT is performed with a combination of electrophysiology tests and MR-DTI. Accordingly, it is vitally important that the location of the lesion in the TG be determined by MR-DTI to ensure adequate puncture for PSR [15]. Consequently, the characteristics of the TGT could serve as a novel indicator of puncture difficulty in PSR, as accurate puncture and treatment are important to ensuring the effects of PSR [7].

Here, we investigated the characteristics of the TGT and the number of punctures needed to perform PSR and observed the effect of novel customized PSR approaches guided by combining MR-DTI and 3D-CT skull base reconstruction in TN patients. To our knowledge, this is the first study on the effect of the characteristics of the TGT on the puncture difficulty and treatment efficacy of PSR.

## 2. Methods

### 2.1. Participants

From May 2012 to March 2022, 48 patients with TN underwent PSR treatments at the Department of Neurosurgery. In our observational study, 17 patients who underwent PSR guided by both the TGT and FO and 31 patients who underwent PSR guided by only the FO were included. We compared the puncturing difficulty and treatment effect of PSR before and after selection of the TGT in guiding the PSR approach. This study was approved by the Ethics Committee of the China-Japan Union Hospital of Jilin University, and patients consented to the publication of medical images.

### 2.2. Image Acquisition and Postprocessing

**Figure 1:**
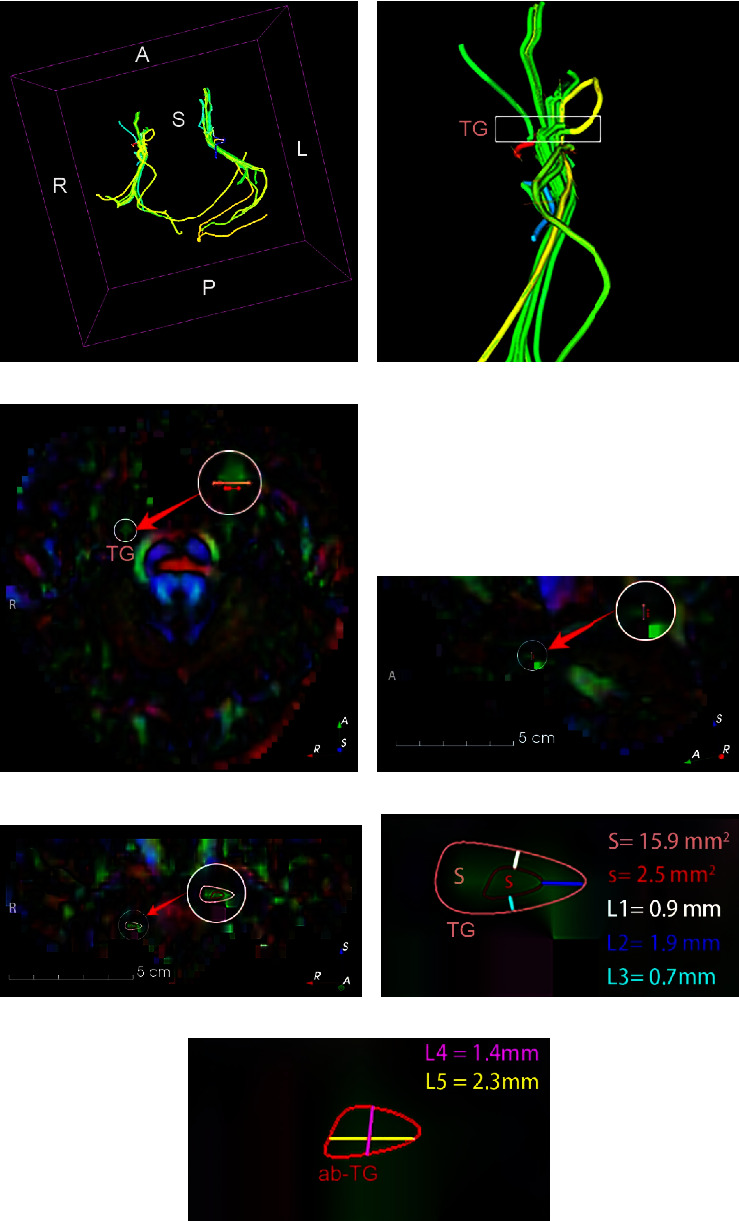
CNV tract and color-FA map including the CNV obtained from MR-DTI. (a) Bilateral CNV tracts in 3D space; (b) the focal segment of the CNV on the affected side; the white box on the affected CNV shows the TG; (c–e) axial, sagittal, and coronal views of the color-FA map, including the affected CNV tracts; insets show the size and position of the TGT (red) and the TG (pink) in the white magnified circle; (f) the position of the TGT on a coronal section of the TG; (g) the size (long and short diameters) of the TGT on a coronal view of the color-FA map.

**Figure 2:**
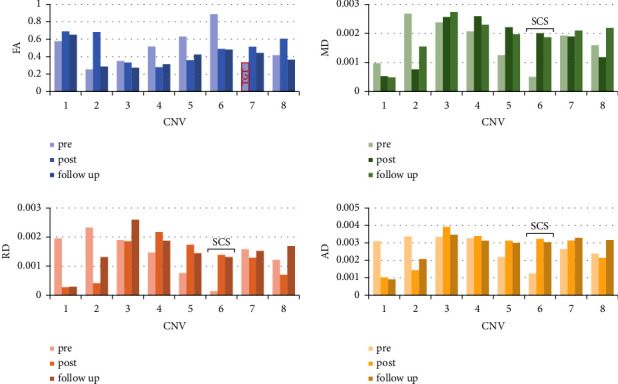
Bar charts of the diffusivity metrics (FA, MD, RD, and AD) of the right CNV obtained from preoperative, postoperative, and follow-up MR-DTI. Images show the diffusivity metrics (FA, MD, RD, and AD) of the affected CNV on preoperative, postoperative, and follow-up MR-DTI according to the segmentation of the trigeminal nerve and their corresponding changes across these time points. The red line represents the TGT and SCS in the CNV segmentation. TGT: trigeminal ganglion target; SCS: significant change segment.

The patients underwent preoperative MR-DTI and/or 3D-CT scans (DTI: spin‒echo echo-planar imaging sequence, 72 directions; in-plane voxel size, 2 × 2; slice thickness, 2 mm; echo time, 95 ms; repetition time, 4100 ms; 3.0 Tesla Siemens MR scanner). The MR-DTI data were postprocessed in the 3D Slicer V4.11 software package utilities (https://www.slicer.org/). We applied tractography to delineate the bilateral CNVs on DTI and calculated the scalar maps (fractional anisotropy (FA), mean diffusivity (MD), radial diffusivity (RD), and axial diffusivity (AD)) (Figure 1). Then, we extracted the diffusivity metrics from the 8 manually delineated segments of the CNV tracts according to the slice thickness of the coronal images (Figure 2). According to previous literature, the FA values of the affected side were lower than those of the contralateral side [12–14]. We defined the significantly low FA segment of the TG as the therapeutic target of PSR, in other words, the TGT. The position and size of the TGT are marked on a coronal image of the FA map in Figure 1.

**Figure 3:**
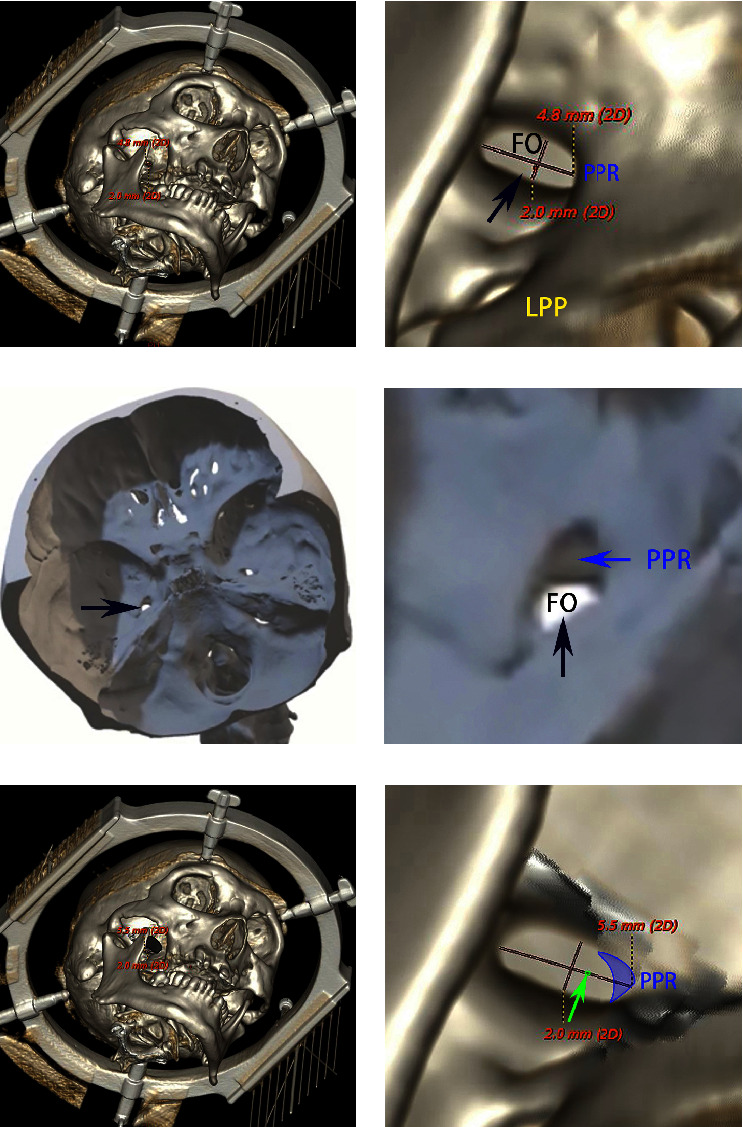
Three-dimensional computed tomography reconstruction images in the operational view. (a) The transverse diameter of the FO was 4.8 mm, and the anterior-posterior diameter was 2.0 mm. The image on the right shows a magnification of the FO from the left. (b) A 3D-printed model shows the shape of the FO and PPR from an interior view of the skull base. (c) We removed part of the PPR obstructing the FO through the mandibular notch, ensuring the integrity of the FO boundary. The transverse diameter was changed to 5.5 mm. The image on the right shows a magnification of the FO from the left.

The 3D reconstruction of the skull base on 3D-CT was used to identify the characteristics of the FO (GE AW 4.6 Image Postprocessing Workstation, GE Healthcare). For an illustrative case, 3D-CT skull base reconstruction images and a 3D-printed model of the patient demonstrated the difficulty of puncture: obfuscation of the FO by the pterygoid process ridge (PPR) and the lateral pterygoid plate (LPP) and the small size of the FO (Figure 3).

Morphological MRI at the cerebellopontine angle was used to identify intracranial focal lesions and compression of the CNV root on the affected side by the adjacent vessel [16].

### 2.3. Treatment

The operations were performed using Komai's CT stereotactic frame (Mizuho Medical Innovation, Tokyo, Japan). We designed accurate trajectories for PSR according to the characteristics of the TGT and/or FO on the MR-DTI images; in particular, the position and size of the TGT and TG aided in adjusting the arc angle of the probe. Then, we performed PSR guided by the TGT for 17 TN patients and by the FO for 31 TN patients [8, 9, 17]. During the operation, we performed radiofrequency treatment and electrophysiology tests. The voltage of the electrophysiology sensory test ranged from 0.15 to 0.30 V, and the voltage of the motor test ranged from 0.9 to 1.2 V. Next, we performed 120 seconds of continuous radiofrequency ablation with a temperature ranging from 65 to 72°C.

### 2.4. Evaluation of the Treatment Effect of PSR

The effect of PSR treatment was evaluated during the postoperative and follow-up periods. The treatment outcome of the TN patients was assessed by a visual analog scale (VAS), on the MR-DTI, and in terms of complications.

### 2.5. Statistical Analysis

Summary statistics were computed for all data using SPSS v26.0 (IBM, USA) in this study. The data were compared between the TGT- and FO-guided PSR groups using the independent-sample*t* test. The correlation between the TGT characteristics and the number of PSR punctures was determined with a 2-tailed Pearson's correlation (*P*  <  0.05). In all statistical tests, *P*  <  0.05 was considered statistically significant.

## 3. Results

### 3.1. Characteristics of TN Patients Who Underwent PSR

**Figure 4:**
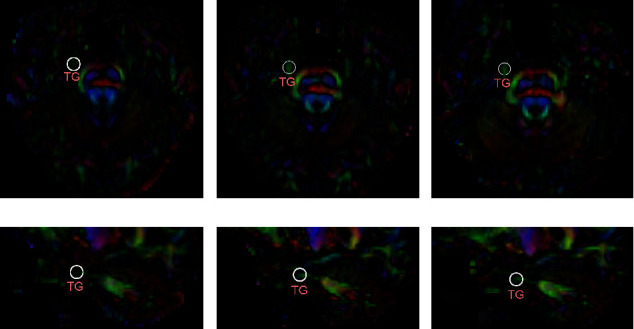
Axial and sagittal views of the color-FA map including the TG obtained preoperatively, postoperatively, and at follow-up. The image shows axial and sagittal views of the color-FA map, including the affected CNV, preoperatively (a, d), 2 days postoperatively (b, e), and at the 4-month follow-up (c, f). The white circle indicates the TG.

The baseline characteristics of the 48 TN patients are summarized in Table 1. Compared with that of the contralateral CNV, the FA of the segment of the TG on the affected side was interrupted and therefore lower, identifying an abnormal segment (Figure 4).

### 3.2. Number of PSR Punctures for the TN Patients

**Figure 5:**
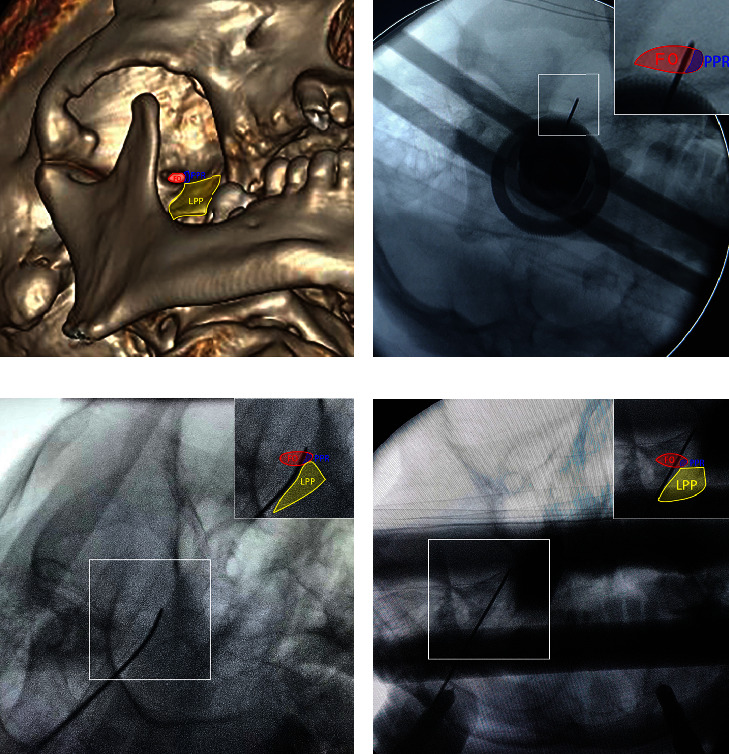
Probe placement into the FO confirmed by C-arm radiography. (a) Three-dimensional computed tomography reconstruction images show the anatomical adjacency among the FO (red shadow), PPR (blue shadow), and LPP (yellow shadow). (b) The first puncture into the FO shows that the probe reached the FO successfully and that the probe was straight. (c) The second puncture to the FO. We decreased the arc angle, and the probe was curved due to being obscured by the PPR. (d) The third puncture to the FO. We increased the arc angle to reach the TGT with the probe through the FO.

Among the patients who underwent PSR guided by the TGT, only one patient (the illustrative case) required three punctures to successfully target the TGT; the other patients only required a single puncture each. The three punctures in the illustrative case all reached the FO target, as confirmed by intraoperative C-arm X-ray (Figure 5). The first puncture to the TG controlled the V2 region, as confirmed by an electrophysiology test. Then, we reached the TGT successfully after 2 adjustments and confirmed that the probe covered the pain territory (V1) accurately with another electrophysiology test.

Among patients who underwent PSR guided by the FO, four patients required multiple punctures, including two patients who underwent two punctures and another two patients who required three punctures.

### 3.3. Characteristics of the TGTs in TN Patients

**Figure 6:**
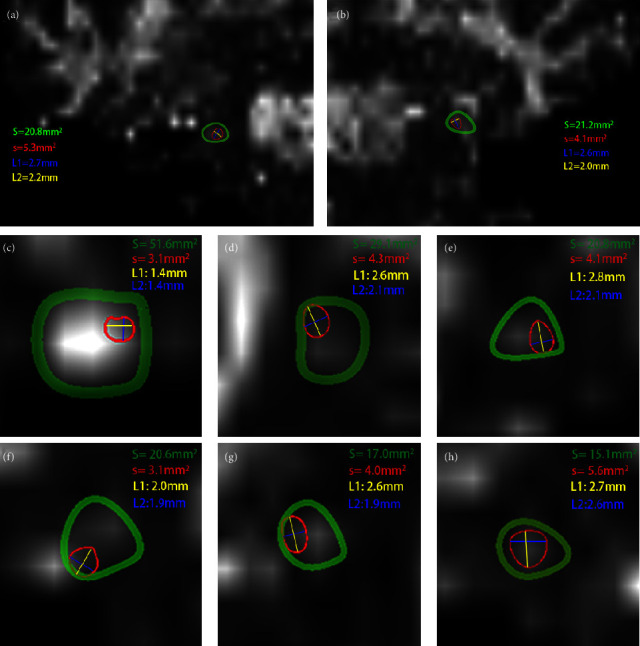
Characteristics of the TGTs in the coronal view of FA maps. The TGTs were located in the center (h), right edge (b, g), lower right (f), upper left (c), lower left (a, e), and upper right of the TG (d). Green circle: TG; Red circle: TGT; S: area of the TG; s: area of the TGT; L1: long diameter of the TGT; L2: short diameter of the TGT.

The size and position of the TGTs within the TG varied with the coronal view of the FA map (Figure 6; Table 2). The position of TGTs in TG also differed. In 17 patients who underwent PSR guided by the TGT, 3 TGTs (17.6%, 3/17) were located in the center of the TG, and the other 14 TGTs (82.4%, 14/17) were found in different positions, including 1 at the upper right, 2 at the right edge, 5 at the lower right, 1 at the upper left, 4 at the lower left, and 1 at the lower edge.

In the illustrative case, the size of the TGT on the coronal view was the smallest at 2.5 mm^2^, and the short diameter of the TGT was 1.4 mm. The TGT was located laterally and superiorly in the TG. The patient was a 74-year-old male who presented with right-sided V2 and V1 TN. The pain mainly spanned from the upper lip and near the nose.

### 3.4. Correlation of the Characteristics of the TGTs with the Number of PSR Punctures

In analyzing the data of patients who underwent PSR guided by the TGT (Table 3), the size of the TGT was significantly negatively correlated with the number of PSR punctures (*r* = −0.6, *P*  <  0.05), and the short diameter of the TGT was significantly negatively correlated with the number of PSR punctures (*r* = −0.6, *P*  <  0.05).

### 3.5. The Effect of PSR on TN Patients

In patients who underwent PSR guided by the TGT, the VAS score decreased from 10 to 1 in 15 patients (88.2%) at 1 day postoperatively and to 4 in the other 2 patients (11.8%) (Table 4). The VAS score decreased to 1 for all patients at 10 days postoperatively. There were no common complications in any of the 17 patients, and only one patient (5.9%) presented with a rare complication of apnea.

In the illustrative case, there were no postoperative complications. On the first postoperative day, the VAS score in the V2 control area was 1, while that in the V1 control area was 4. At 2 days postoperatively, the VAS score had decreased to 1, and the FA of the TGT was increased (Figure 2). At a 3-monthfollow-up, the VAS score of the patient was 1, and the medication was suspended. During the 4-month follow-up, the patient underwent an MR-DTI scan, and the FA of the TGT was greater than the preoperative value (Figure 2).

In patients who underwent PSR guided by the FO, the VAS score decreased from 10 to below 3 in 28 patients (90.3%), to 4 in one patient (3.2%), and to 7 in two patients (6.5%) 1 day after the operation (Table 4). Two patients (6.5%) still had a VAS score greater than 3 at 10 days postoperatively. Seven patients (22.6%) experienced facial numbness after treatment, but this side effect subsided within six months after surgery. One patient (3.2%) experienced a hematoma of the facial soft tissue, and one patient (3.2%) experienced residual facial pain. Four patients (12.9%) experienced pain recurrence 1 to 2 years after surgery.

There was no significant difference in the VAS score between the two TN patient groups (*P*  >  0.05). However, the rate of complications in the PSR guided by the FO group was higher than that in the PSR guided by the TGT group.

## 4. Discussion

We investigated the effect of the characteristics of the TGT on radiofrequency treatment in TN patients in our study. The results suggest that these characteristics are correlated with the number of punctures required to perform PSR and are beneficial for guiding radiofrequency treatment. The characteristics of the TGT may be a valuable predictive indicator of the difficulty of puncture in PSR. In particular, patients with small TGTs will likely need multiple punctures. The PSR approach has two key steps for TN treatment: localization of both the FO and TG.

First, the puncture probe passes through the FO; this structure is difficult to access successfully with a single puncture. Guo et al. reported that the puncture failure rates range from 10% to 20% for experienced neurosurgeons [8]. It was previously reported that 2–4% of the FOs of TN patients are difficult to access, and multiple unsuccessful punctures may induce complications [17, 18]. Many recent studies have reported on the difficulty in puncturing the FO for TN treatment, and thus this difficulty has become widely acknowledged. A variety of factors influencing this difficulty have been reported, including (1) whether the trajectory is obstructed by the maxilla or mandible or if the distance between the mandible and tooth ridge is too narrow; (2) whether the FO is covered by the LPP and/or the PPR; and (3) the size of the FO, which, due to anatomic variability, can be small [9, 19].

The problem of the difficult puncture of the FO during the operations is a subject of seemingly constant investigation. To avoid the influence of soft tissue in easily displacing the probe in the Härtel route, Matthew et al. reported that bony landmarks should be identified during puncture [20]. Bony landmarks are easy to identify and easily marked on the surface of the face and visualized in X-ray projections. Various techniques and applications, such as 3D-CT skull base reconstruction, stereotactic frames, or navigation, have been implemented to resolve these issues [1, 7]. The aforementioned studies suggested that accurate puncture allows for a more efficient operation and a reduced need for repeat punctures. The operative plan is tailored so that we can determine the design of the trajectory, identify the best operative position, and predict intraoperative difficulties.

Second, it is well known that the FO is just the first target of PSR, as the probe also needs to reach the TG after passing through the FO. Generally, for patients for whom the V1 or V2/V3 branch is affected, the inner third or half of the transverse diameter of the FO is selected as the puncture target [9]. However, the TGT is the ideal but invisible puncture target in PSR. Specific parts of the TG control the V1, V2, and V3 branches of CNV, but the location of the actual therapeutic TGT has not been identified previously. In our novel model, we used MR-DTI to identify the specific part of the TG responsible for the affected branches and named it the TGT. Then, we designed the operation scheme with guidance by the TGT and FO, which assisted in accurately performing PSR at the TGT. Especially in the illustrative case, the area of the TGT was 2.4 mm^2^, and its shorter diameter was only 1.4 mm. Nonetheless, the diameter of the puncture probe is 0.9 mm, which makes it challenging to successfully puncture the TGT. Therefore, the characteristics of the TGT are strongly correlated with the number of punctures in the PSR; that is, a small TGT size may be a valuable predictive indicator for the need for multiple punctures.

The success of the TGT puncture is important to ensure the effect of PSR. The TG is located at the anterior, inferior, and lateral sides of Meckel's cave in the skull base, and the ganglion is small due to its concave shape [21–23]. The difficulty in accessing TGT was assessed by the combination of 3D-CT and MR-DTI. When the probe has passed through the FO, it can be inserted into the part of the TG that projects to the affected branches. The probe location can influence the efficacy and complications of the therapy [15, 23]. In practice, the parts of the TG that project to the affected branches usually need to be validated by an electrophysiology test [1]. However, a high rate of complications due to radiofrequency treatment, such as facial numbness, facial paresthesia, masseter weakness, decreased corneal reflex, and keratitis, has also been observed [1, 24]. In our study, the effect of treatment between the two groups was not significantly different, but postoperative complications were less common in the group that underwent PSR guided by the TGT. The model primarily highlights the significance of puncturing the TGT during PSR for achieving fewer complications.

However, we used MR-DTI to detect the TGT for predicting the difficulty of puncture in PSR, and the novel model, combining 3D-CT reconstruction and MR-DTI, improved the accuracy, safety, and efficiency of PSR for TN patients.

Limitations: at present, we are not yet able to precisely fuse CT and MRI, making it impossible to measure the precise distance and angle of the TGT puncture. Additionally, this study is only an exploratory study with a small sample size, and further study with a larger sample size is needed. However, this study also does not involve an overall sampling problem and lacks underlying data, so no sample size calculation can be performed. In our observational study, the sample size was the number of patients who had FO and/or TGT-guided PSR procedures applied.

## 5. Conclusions

We found that the characteristics of the TGT were correlated with the number of punctures in the PSR. The application of MR-DTI for detecting the size of the TGT is an important step in predicting the difficulty of puncture. The PSR approach can be guided by TGT and FO for TN patients who have multiple factors affecting the difficulty of puncture, which may be beneficial to producing a smaller number of complications.

## Figures and Tables

**Table 1:** TN patient characteristics.

	PSR guided by the TGT	PSR guided by the FO
Total	17	31
Female	8	13
Male	9	18
Age range	38–85	52–78
Affected side		
Left	5	6
Right	12	25
Affected branches		
V1	1	2
V2	6	10
V3	2	6
V1 and V2	4	2
V2 and V3	2	9
V1, V2 and V3	2	2

**Table 2:** Characteristics of the TGT and TG (*n* = 17).

	MIN	MAX	First quartile	Median	Third quartile	95% CI	Mean ± SD
S (mm^2^)	15.4	51.6	20.5	24.6	29.1	(9.3, 41.3)	25.1 ± 8.2
s (mm^2^)	2.5	5.4	4.0	4.4	4.9	(2.7, 5.8)	4.3 ± 0.8
L1 (mm)	2.0	3.0	2.5	2.6	2.8	(2.1, 3.1)	2.6 ± 0.3
L2 (mm)	1.4	2.4	1.9	2.0	2.2	(1.5, 2.6)	2.0 ± 0.3
s/S (%)	6.0	28.6	15.0	18.3	19.8	(7.7, 28.4)	18.2 ± 5.3

S: area of the TG; s: area of the TGT; L1: long diameter of the TGT; L2: short diameter of the TGT; s/S: ratio of the area of the TGT to that of the TG; 95% CI: 95% confidence interval for the mean (lower and upper limits).

**Table 3:** Correlation between characteristics of the TGT and number of punctures in PSR (*n* = 17).

No.	TGs (mm^2^)	TGTs (mm^2^)	L1 (mm)	L2 (mm)	TGT/TG (%)	Punctures
1	20.6	3.1	2	1.9	15.1	1
2	22.4	4.6	2.8	2	20.5	1
3	29.3	4.4	3	1.9	15.0	1
4	51.6	3.1	2.2	1.4	6.0	1
5	19	5	2.5	2.1	26.3	1
6	15.9	2.5	2.3	1.4	15.7	3
7	27.2	5	2.8	2.4	18.4	1
8	15.4	4.4	2.7	2.2	28.6	1
9	29.1	4.3	2.6	2.1	14.8	1
10	26.6	4.7	2.6	2.3	17.7	1
11	29.4	5.4	3	2.3	18.4	1
12	29	4	2.4	2	13.8	1
13	24.6	4.5	2.5	2.2	18.3	1
14	24.8	4	2.6	1.9	16.1	1
15	20.4	3.9	2.5	1.9	19.1	1
16	20.8	5.3	2.7	2.2	25.5	1
17	21.2	4.1	2.6	2	19.3	1

**Table 4:** Effects of PSR on TN patients.

	PSR guided by the TGT	PSR guided by the FO
Immediate efficacy	15 (88.2%)	28 (90.3%)
Follow-up efficacy	17 (100%)	24 (77.4%)
Complications	1 (5.9%, apnea)	2 (6.4%)
Recurrences	0	4 (12.9%)

## Data Availability

The data used to support the findings of this study are included within the supplementary information files.

## Supplementary Materials

Supplementary Materials The supplementary material for this article is included within the supplemental file. Supplementary 1: STROBE checklist; Supplementary 2: CONSORT checklist; Supplementary 3: the data used to support the findings of this study included the relevant raw data tables and the results of the statistical analysis of the data.
