# Supplementary material for: Effects of the MR-DTI Characteristics of the Trigeminal Ganglion Target on Radiofrequency Treatment in Patients with Trigeminal Neuralgia: A Retrospective Observational Clinical Study
Source: Pain Res Manag. 2023 Jan 17;2023:1988926. doi: 10.1155/2023/1988926 (PMC10266916; doi:10.1155/2023/1988926)
Supplement: Supplementary Materials — The supplementary material for this article is included within the supplemental file. Supplementary 1: STROBE checklist; Supplementary 2: CONSORT checklist; Supplementary 3: the data used to support the findings of this study included the relevant raw data tables and the results of the statistical analysis of the data. [file 1988926.f1.zip › data-1.rtf]

NO.	affected	TGT Segment	TGs	TGTs	L1	L2	TGT/TG	puncture	
1	V2	5	20.6	3.1	2	1.9	0.1505	1	
2	V1, V2, V3	6	22.4	4.6	2.8	2	0.2054	1	
3	V1, V2	7	29.3	4.4	3	1.9	0.1502	1	
4	V1	7	51.6	3.1	2.2	1.4	0.0601	1	
5	V2, V3	7	19	5	2.5	2.1	0.2632	1	
6	V1, V2	7	15.9	2.5	2.3	1.4	0.1572	3	
7	V1, V2	7	27.2	5	2.8	2.4	0.1838	1	
8	V2	6	15.4	4.4	2.7	2.2	0.2857	1	
9	V2, V3	4	29.1	4.3	2.6	2.1	0.1478	1	
10	V1, V2, V3	5	26.6	4.7	2.6	2.3	0.1767	1	
11	V3	6	29.4	5.4	3	2.3	0.1837	1	
12	V2, V1	6	29	4	2.4	2	0.138	1	
13	V2	6	24.6	4.5	2.5	2.2	0.183	1	
16	V1,V2,V3	6	23.7	4.2	2.5	2	0.177	1	
14	V2	5	24.8	4	2.6	1.9	0.16129	1	
15	V3	4	20.4	3.9	2.5	1.9	0.191176	1	
17	V2	6	20.8	5.3	2.7	2.2	0.254808	1	
1. Raw measurements of TG and TGT characteristics in DTI images in 17 patients

2. Descriptive statistical results of the raw data
	TG	TGT	L1	L2	TGT/TG	puncture	
MIN	15.4	2.5	2	1.4	0.0601	1	
MAX	51.6	5.4	3	2.4	0.2857	3	
MD	25.28235	4.258824	2.570588	2.011765	0.180563	1.117647	
SD	8.147257	0.788241	0.261641	0.278124	0.052629	0.485071	
MEDIAN	24.6	4.4	2.6	2	0.177	1	
1/4	20.5	3.95	2.45	1.9	0.15035		
3/4	29.05	4.85	2.75	2.2	0.198288	1	
95% upper limitation	9.31373	2.713872	2.057772	1.466642	0.07741	0.166907	
96% lower limitation	41.25098	5.803775	3.083404	2.556888	0.283717	2.068387	
 

Correlation

Descriptive statistics	
	Mean	Std	No.	
TGTs	4.26	.788	17	
numbers	1.12	.485	17	


Analysis of correlation	
	TGTs	numbers	
TGTs	Pearson correlation	1	-.575*	
	Sig.¡]Two-tailed¡^		.016	
	No.	17	17	
numbers	Pearson correlation	-.575*	1	
	Sig.¡]Two-tailed¡^	.016		
	No.	17	17	

*. At the 0.05 level (two-tailed), correlation significant. 	


Correlation
Descriptive statistics	
	Mean	Std	No.	
numbers	1.12	.485	17	
L2	2.01	.278	17	

Analysis of correlation	
	numbers	L2	
numbers	Pearson correlation	1	-.567*	
	Sig.¡]Two-tailed¡^		.018	
	No.	17	17	
L2	Pearson correlation	-.567*	1	
	Sig.¡]Two-tailed¡^	.018		
	No.	17	17	


*. At the 0.05 level (two-tailed), correlation significant. 	

guided by FO	VAS	puncture	
1	1	1	
2	1	1	
3	1	1	
4	1	2	
5	1	1	
6	1	1	
7	2	1	
8	1	1	
9	2	1	
10	1	2	
11	1	1	
12	1	1	
13	4	3	
14	1	1	
15	1	1	
16	1	1	
17	1	1	
18	1	1	
19	7	3	
20	1	1	
21	1	1	
22	2	1	
23	1	1	
24	1	1	
25	7	1	
26	1	1	
27	1	1	
28	1	1	
29	1	1	
30	1	1	
31	1	1	
guided by TGT	VAS	
1	M	1	
2	M	1	
3	F	1	
4	M	1	
5	F	1	
6	F	1	
7	M	1	
8	F	4	
9	M	1	
10	M	4	
11	F	1	
12	F	1	
13	M	1	
14	F	1	
15	M	1	
16	M	1	
17	F	1	
3. Number of punctures in PSR and VAS score data for both groups


T-Test
	

Group statistics	
	Group	N	Mean	Std.	Std. error mean	
VAS1	1	17	1.35	.996	.242	
	2	31	1.58	1.566	.281	


	Levene's Test for Equality of Variances	t-test for Equality of Means	
	F	Sig.	t	df	Sig.¡]2-tailed¡^	Mean Difference	
							
VAS1	Equal variances assumed	.893	.350	-.541	46	.591	-.228	
	Equal variances not assumed			-.614	44.831	.542	-.228	


4. Basic demographic information of patients in both groups
	Patients (guided by TGT)	Patients (guided by FO)	
Total 	17	31	
Female	8	13	
Male	9	18	
Age range	38-85	52-78	
Affected side		
Left	5	6	
Right	12	25	
Affected divisions	
V1	1	2	
V2	6	10	
V3	2	6	
V1, V2	4	2	
V2, V3	2	9	
V1, V2, V3	2	2	
